# The value of intraoperative intracranial pressure monitoring for predicting re-operation using salvage decompressive craniectomy after craniotomy in patients with traumatic mass lesions

**DOI:** 10.1186/s12893-015-0100-7

**Published:** 2015-10-14

**Authors:** He-xiang Zhao, Yi Liao, Ding Xu, Qiang-ping Wang, Qi Gan, Chao You, Chao-hua Yang

**Affiliations:** Department of Neurosurgery, West China Hospital, Sichuan University, No. 37 Guoxue Xiang, Chengdu, Sichuan 610041 P. R. China; Department of Neuro-intensive care unit, West China Hospital, Sichuan University, No. 37 Guoxue Xiang, Chengdu, Sichuan 610041 P. R. China

**Keywords:** Decompressive craniectomy, Severe traumatic brain injury, Intracranial pressure, Re-operation, Mass lesion

## Abstract

**Background:**

The risk factors of predicting the need for postoperative decompressive craniectomy due to intracranial hypertension after primary craniotomy remain unclear. This study aimed to investigate the value of intraoperative intracranial pressure (ICP) monitoring in predicting re-operation using salvage decompressive craniectomy (SDC).

**Methods:**

From January 2008 to October 2014, we retrospectively reviewed 284 patients with severe traumatic brain injury (STBI) who underwent craniotomy for mass lesion evacuation without intraoperative brain swelling. Intraoperative ICP was documented at the time of initial craniotomy and then again after the dura was sutured. SDC was used when postoperative ICP was continually higher than 25 mmHg for 1 h without a downward trend. Univariate and multivariate analyses were applied to both initial demographic and radiographic features to identify risk factors of SDC requirement.

**Results:**

Of 284, 41 (14.4 %) patients who underwent SDC had a higher Initial ICP than those who didn’t (38.1 ± 9.2 vs. 29.3 ± 8.1 mmHg, *P* < 0.001), but there was no difference in ICP after the dura was sutured. The factors which have significant effects on SDC are higher initial ICP [odds ratio (OR): 1.100, 95 % confidence interval (CI): 1.052–1.151, *P* < 0.001], older age (OR: 1.039, 95 % CI: 1.002–1.077, *P* = 0.039), combined lesions (OR: 3.329, 95 % CI: 1.199–9.244, *P* = 0.021) and early hypotension (OR: 2.524, 95 % CI: 1.107–5.756, *P* = 0.028). The area under the curve of multivariate regression model was 0.771.

**Conclusions:**

The incidence of re-operation using SDC after craniotomy was 14.4 %. The independent risk factors of SDC requirement are initial ICP, age, early hypotension and combined lesions.

## Background

Including contusions and subdural haematomas (SDHs), more than 50 % cases of severe traumatic brain injury (STBI) are associated with mass lesions [1, 2]. The vicious cycle of mechanical compression, oedema, hypoxia and ischemia leads to rapidly deteriorating secondary injuries which require urgent surgical intervention. However, after mass lesion evacuation, the excised bone flap presents a problem for surgeons, particularly when patients do not have obvious fulminant brain swelling [3]. After craniotomy, repositioning the bone flap can cause postoperative intracranial hypertension, and the surgeon may eventually need to perform re-operation using salvage decompressive craniectomy (SDC). On the other side, primary decompressive craniectomy (DC) can further decrease intracranial pressure (ICP) and attenuate the probability of re-operation in subsequent treatment. Even so, DC is questioned by the uncertain outcomes and the frequent complications [4, 5]. It is believed that DC is performed for the purpose of saving lives but at a price of severe disability in return [6]. While combining the benefits of DC and the risks of craniotomy, the final decision primarily depends on the experience of the surgeon.

ICP monitoring has been generally approved in evidence-based guidelines for patients with STBI [7, 8]. However, no matter what the treatment strategies or the prognosis tools derived from ICP or cerebral perfusion pressure (CPP) monitoring are, current studies only focus on postoperative or conservative patient care [9–13]. As an objective indicator, if intraoperative ICP could provide valuable information about the risks of craniotomy, it would be helpful to avoid unnecessary injury and save limited medical resources.

There is not sufficient clinical evidence to allow for an unbiased treatment choice for STBI [14]. There are some notable ongoing clinical trials, such as RESCUE-ICP and RESCUE-ASDH [15, 16]. In our centre, according to the philosophy of minimal unnecessary damage, we choose only primary DC in cases of intraoperative brain swelling during initial surgery. In this study, according to intraoperative ICP variations, we assess the risk for the need of SDC after craniotomy in patients with traumatic mass lesions.

## Methods

### Study population

We retrospectively reviewed the records of patients who were treated for STBI with emergency surgery at the Neuro-trauma Centre of West China Hospital, Sichuan University, from January 2008 to October 2014. The study was approved by the West China Hospital Clinical Trials and Biomedical Ethics Committee (NO. 2015–88). Patients who met the following criteria were included in the study: (1) Glasgow Coma Scale (GCS) of ≤ 8, (2) age between 15 and 65 years, (3) Marshall Classification VI, presence of high- or mix-density lesion of ≥ 25 ml (contusion and SDH) and (4) a neurological status of progressive deterioration after injury. Patients who met the following criteria were excluded: (1) bilateral mydriasis of critically endangered status, (2) cerebellar contusion, (3) penetrating brain injury, (4) admission > 24 h after injury, (5) serious extracranial injury with unstable vital signs and (6) definite surgical contraindications.

### Interventions

During preoperative preparation, the best medical treatments were administrated to all patients following the recommendations of The Brain Trauma Foundation guidelines [17]. An intraparenchymal or intraventricular ICP monitor was placed in the lesion side before craniotomy and kept for at least 5 days after surgery. Intraoperative ICP values were documented at the time of craniotomy (initial ICP) and after the dura was sutured (dura suture ICP).

The unilateral trauma craniotomy mode suggested by Potts et al. was preferred [18]. The scalp flap included the frontal, parietal and temporal areas located 1 cm beyond the brim of the skull window. The range of craniotomy was at least 15 × 12 cm, extending down to the temporal base and curving around the parietal lobe to the side within 2 cm of the midline. After dura incision, contused and necrotised tissues were removed. The dura was sutured on expansion by the temporal fascia or artificial dura. The only criterion for primary DC was the development of intraoperative brain swelling which was defined as brain tissue protruding above the base of the temporal skull window, impeding normal repositioning of the bone flap after the mass lesion had been removed.

The treatment was aimed maintaining IC*P* < 20 mmHg and CPP > 60 mmHg. Stepwise control of ICP was provided in the NICU. First-tier therapies included sedation, neuromuscular blockade, intubation, ventilation, 30° head elevation, osmotic dehydration and external ventricular drainage. The second-tier therapy was hypothermia (32 °C–34 °C). SDC was used subsequently if ICP continued to be > 25 mmHg for 1 h without a downward trend even after all other treatments had been attempted.

### Data collection and statistics

The following demographic and clinical features were analysed: age, sex, clinical severity (GCS, motor score and pupillary reaction), CT parameters (mass lesion type, midline shift and compressed basal cisterns), time from injury to operation, intraoperative ICP and reason for re-operation. The Extended Glasgow Outcome Score (GOSE) was assessed after 6 months of surgery by a face-to-face or telephonic interview.

All patients were divided into SDC or non-SDC group according to whether or not SDC was required for re-operation. Univariate analysis was performed between the potential preoperative variables and SDC requirement. Since there was not much previous research of SDC on patients with mass lesions, we referenced the potential variables for the risk of SDC requirement correlating with the studies of postoperative intracranial hypertension and the prognosis models of poor outcome [19–21]. Parametric variables were presented as mean ± standard deviation and analyzed by Student’s *t*-test. Non-parametric variables were presented as a ratio and analysed by Pearson chi-square test. Potential variables with *P* < 0.05 were selected for multivariate logistic regression analysis to identify the independent risk factors. The relationship between potential variables and SDC requirement was assessed in terms of odds ratio (OR) and R^2^. To evaluate the prediction value of the multivariate model, a receiver-operating characteristic (ROC) curve was designed. The goodness-of-fit test was performed using the Hosmer–Lemeshow method [22]. *P* < 0.05 was considered statistically significant. All statistical analyses were computed using SPSS software (version 19.0).

## Results

**Fig. 1:**
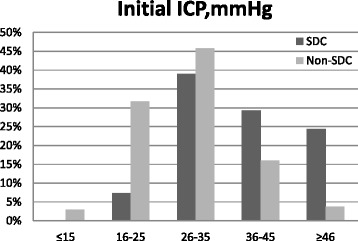
Distribution of initial ICP in the SDC group and non-SDC group

The demographic and clinical characteristics of the patients are shown in Table 1. In total, 284 patients underwent craniotomy for mass lesion evacuation and bone flap repositioning over a period of 7 years. Forty-one (14.4 %) patients underwent SDC for refractory intracranial hypertension. The main reason for re-operation using SDC was regional oedema and progressive traumatic haematomas (82.9 %). The mean GCS was 5.2, ranging from 3 to 8. In the radiological findings of preoperative CT scan, midline shift > 5 mm was observed in 219 (77.1 %) patients. Compressed basal cisterns with close proportion (74.3 %) were observed in 211 patients. SDH was the main type of mass lesion, occurring in 122 (43.0 %) patients. The mean initial ICP in all patients was 30.6 ± 8.7 mmHg, ranging from 13 to 54 mmHg. Figure 1 shows that initial ICP was significantly higher in the SDC group than in the non-SDC group (*P* < 0.001). The mean dura suture ICP was 9.3 ± 3.4 mmHg. We did not find ICP > 16 mmHg in either group after the dura was sutured.

**Table 1:** Patient demographics and clinical characteristics

Characteristics	
Total patient, n	284
SDC, n (%)	41 (14.4 %)
Reason for re-operation, n (%)	
Regional oedema and PTH^a^	34 (82.9 %)
Focal neonatal haematoma^b^	6 (14.6 %)
Remote site haematoma^c^	1 (2.4 %)
Age, years (mean ± SD)	35.4 ± 10.8
Male, n (%)	235 (82.7 %)
Cause of trauma, n (%)	
Traffic accidents	200 (70.4 %)
Fall	54 (19.0 %)
Assault	25 (8.8 %)
Others	5 (1.8 %)
Time from injury to operation, h (mean ± SD)	9.8 ± 4.9
Trauma severity, GCS, n (%)	
3	41 (14.4 %)
4	57 (20.1 %)
5	66 (23.2 %)
6	64 (22.5 %)
7	36 (12.7 %)
8	20 (7.0 %)
CT parameters, n (%)	
Midline shift of >5 mm	219 (77.1 %)
Compressed basal cisterns	211 (74.3 %)
Mass lesion type, n (%)	
Combined lesions^d^	81 (28.5 %)
SDH	122 (43.0 %)
Contusion	81 (28.5 %)
ICP, mmHg (mean ± SD)	
Initial ICP	30.6 ± 8.7
Dura suture ICP	9.3 ± 3.4
Unfavorable outcome^e^, n (%)	193 (68.0 %)

*CT* Computed tomography, *GCS* Glasgow Coma Scale, *ICP* Intracranial pressure, *PTH* Progressive traumatic haematoma, *SD* Standard deviation, *SDC* Salvage decompressive craniectomy, *SDH* Subdural haematoma

^a^Increase more than 30 % volume of original haematoma

^b^Focal neonatal haematoma in the surgical region

^c^Neonatal haematoma away from the surgical region

^d^Contusion plus SDH

^e^Extended Glasgow Outcome Score (1–4)

Over 6 months of follow-up, unfavorable outcomes (GOSE 1–4) were confirmed in 193 (68.0 %) patients. Outcomes were not the principal objectives of the study; nevertheless, we found that both initial ICP (OR: 1.231, *P* < 0.01) and dura suture ICP (OR: 1.187, *P* = 0.01) were significantly correlated with unfavorable outcomes. Higher GCS meant mild severity and lower risk of unfavorable outcomes (OR: 0.322, *P* < 0.001). However, we did not find a significant correlation between SDC requirement and poor outcomes.

**Fig. 2:**
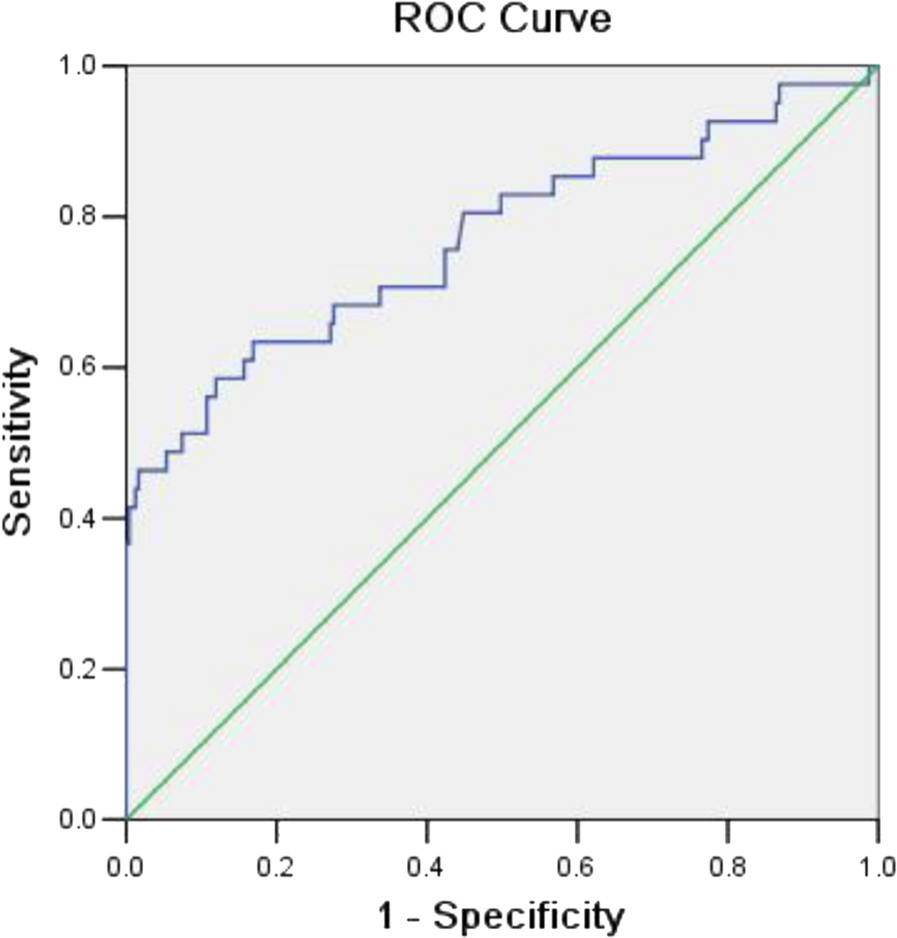
ROC curve of the multivariate analysis model. The area under the curve is 0.771

On screening 18 candidate variables, Table 2 presents potential correlations between SDC requirement and older age, combined lesions, SDH, lower blood pressure and higher initial ICP. By multivariate logistic regression analysis, the independent risk factors are indentified in Table 3. For every 1-mmHg increase in initial ICP, the risk for the need of SDC increased by 10 % [OR: 1.100; 95 % confidence interval (CI): 1.052–1.151]. The Hosmer–Lemeshow test result (*P* = 0.434) indicated that the regression model was well calibrated. SDC could be explained by this model (Nagelkerke R^2^) in 31 % cases. Figure 2 shows the ROC curve established by multivariate analysis. The area under the curve was 0.771 (*P* < 0.001, 95 % CI: 0.678–0.864).

**Table 2:** Univariate analysis of re-operation using SDC

Variables	SDC	non-SDC	*P*	OR (95 % CI)	R^2^
Age, years (mean ± SD)	40.1 ± 11.9	34.7 ± 10.4	0.003	1.048 (1.015–1.081)	0.031
Male, n (%)	32 (78.0 %)	203 (83.5 %)	0.389		
Cause of trauma, n (%)					
Traffic accident	26 (63.4 %)	174 (71.6 %)	0.198		
Fall	11 (26.8 %)	43 (17.7 %)	0.151		
Assault	3 (7.3 %)	22 (9.1 %)	0.717		
Others	1 (2.4 %)	4 (1.6 %)	0.723		
Trauma severity					
GCS score, n (%)			0.802		
3–5	24 (58.5 %)	140 (57.6 %)			
6–8	17 (41.5 %)	103 (42.4 %)			
Motor score, n (%)			0.291		
1–3	29 (70.7 %)	157 (64.6 %)			
4–6	12 (29.3 %)	86 (35.4 %)			
Abnormal pupillary response, n (%)	30 (73.2 %)	164 (67.5 %)	0.471		
CT parameters, n (%)					
Midline shift of >5 mm	33 (80.5 %)	186 (76.5 %)	0.579		
Compressed basal cisterns	32 (78.0 %)	177 (72.8 %)	0.485		
Type of mass lesion, n (%)					
Combined lesions	22 (53.6 %)	59 (24.3 %)	<0.001	3.611 (1.829–7.130)	0.047
SDH	12 (29.3 %)	110 (45.3 %)	0.059	0.500 (0.244–1.026)	0.013
Contusion	7 (17.1 %)	74 (30.4 %)	0.085		
Early hypotension, n (%)	20 (48.8 %)	40 (16.5 %)	<0.001	4.833 (2.400–9.733)	0.064
Time from injury to operation, h (mean ± SD)	10.4 ± 5.6	9.6 ± 4.7	0.362		
ICP, mmHg (mean ± SD)					
Initial ICP	38.1 ± 9.2	29.3 ± 8.1	<0.001	1.125 (1.078–1.174)	0.116
Dura suture ICP	9.4 ± 4.0	9.2 ± 3.3	0.721		
Unfavorable outcome, n (%)	32 (78.0 %)	161 (66.3 %)			

*CI* confidence interval, *CT* Computed tomography, *GCS* Glasgow Coma Scale, *ICP* Intracranial pressure, *OR* Odds ratio, *SD* Standard deviation, *SDC* Salvage decompressive craniectomy, *SDH* Subdural hematoma

**Table 3:** Multivariate logistic regression

Variables	*P*	OR (95 % CI)
Age	0.039	1.039 (1.002–1.077)
Combined lesions	0.021	3.329 (1.199–9.244)
SDH	0.647	
Early hypotension	0.028	2.524 (1.107–5.756)
Initial ICP	<0.001	1.100 (1.052–1.151)

*CI* Confidence intervals, *ICP* Intracranial pressure, *OR* Odds ratio, *SDH* Subdural haematoma

Nagelkerke 0.311, Hosmer–Lemeshow test *P* = 0.434

## Discussion

Correct indication is an inevitable critical issue when we discuss the role of craniotomy or primary DC in STBI treatment. We attempted to evaluate if there is any ideal perspicuous boundary between craniotomy and primary DC and if there is, whether we can quantify the criteria by objective factors. Several authors have tried to evaluate the efficacy of primary DC for STBI treatment in animal models and clinical settings [23–25]. In retrospective studies, other authors established opposing views, indicating that initial DC does not show any significant advantage compared with craniotomy [26, 27]. However, irrespective of the opinion about DC, as the most widely used surgical treatment, craniotomy has become the costar for DC study. One of the limitations of using craniotomy as the initial surgery for STBI due to mass lesions is the probability of re-operation using SDC on account of postoperative deteriorating brain oedema and progressive intracranial hemorrhage.

In this retrospective study, we summarised the characteristics of SDC after craniotomy. The incidence of SDC in our centre was 14.4 %. In the study by Compagnone et al., 11 % patients underwent initial craniotomy and salvage operations [1]. Considering that their subjects included patients undergoing both craniotomy and DC, the actual incidence of re-operation may be higher. Huang et al. reported that 37.5 % patients required re-operation using SDC in the craniotomy group [28]. In our study, the main cause of re-operation was regional oedema and progressive traumatic haematomas (82.9 %) rather than focal neonatal haematomas or remote site haematomas. This explains why we used DC instead of craniotomy for re-operation to decrease refractory intracranial hypertension and evacuate focal lesions.

Our study demonstrated that initial ICP is an independent risk factor of SDC requirement. Intracranial hypertension is the result of various pathophysiological mechanisms and the reason for subsequent intracranial deterioration. Based on ICP monitoring for postoperative and conservative patient care, some neurosurgery guidelines and consensus have been established for rethinking the techniques. Only a few studies have noted the value of intraoperative ICP. Yuan et al. found that initial ICP has a significant prognostic value for intracranial hypertension in diffusion traumatic brain injuries [29]. Kuo et al. concluded that after haematomas evacuation, patients with intraoperative ICP of <17.5 mmHg would have better outcomes [30]. Tsai et al. considered that the ICP threshold for favourable outcomes should be lowered to <14 mmHg [31]. To the best of our knowledge, our study is the first to evaluate the correlation between intraoperative ICP and subsequent salvage re-operation. Use of intraoperative ICP as the main indicator of evaluation was associated with the characteristics of ICP itself. First, ICP can be determined before the final decision of bone flap disposal in the procedure timeline. Second, it is an objective parameter that can directly weigh the clinical event compared with many other demographic and dichotomous imaging variables. Third, it is easy to use. Our data showed that the average initial ICP in the SDC group was almost 10 mmHg higher than that in the non-SDC group (Table 2). According to multivariate regression analysis, initial ICP increments of 10 mmHg would elevate the risk for the need of SDC 1.6 times. Contrary to the results of initial ICP, we did not find any definite correlation between dura suture ICP and SDC requirement. The maximum dura suture ICP did not increase over 16 mmHg in either group. This indicated that through craniotomy, intracranial hypertension was ameliorated; however, the pathologic damage obviously did not cease by the end of surgery. Some studies have indicated that even if the intracranial hypertension is temporarily reversed, ischemic insult and cerebral oedema could occur subsequently [24, 32].

Multivariate regression analysis indicated that higher age, early hypotension and combined lesions were the independent risk factors associated with SDC requirement. With respect to age, the risk for the need of SDC increased by 3.9 % for every 1-year increase in age (OR: 1.039; 95 % CI: 1.002–1.077). This was consistent with the results of previous studies that reported an age-related increase in risk of additional hemorrhage and brain swelling for degeneration of cerebral microstructure [33, 34]. In our study, to ensure competent physiological background and sound extracranial organ function, we did not include patients >65 years. Considering the increased space owing to cerebral atrophy, we believe that DC would be more complicated in geriatrics. In the presence of early hypotension in patients with traumatic mass lesion, the OR of SDC requirement increased 1.52 times (OR: 2.524; 95 % CI: 1.107–5.756). Low systolic blood pressure (<90 mmHg) can directly aggravate cerebral ischemia and lead to diffuse brain swelling [35]. In our centre, all patients with early hypotension received strict blood pressure management recommended by the guidelines [7]. Obviously, this intervention did not completely erase the negative impact of hypotension. We infer that the pre-hospital duration is a potential contributor to hypotension. Samant et al. concluded that the first 6 h hypotension in STBI correlated with poor outcome [36]. However, we can monitor and manage blood pressure only on arrival of the patient. It is impractical to determine the exact length of pre-hospital hypotension. Another significant factor correlating with SDC requirement was combined lesions (OR: 3.329; 95 % CI: 1.199–9.244). We found that disease progression of patients with combined lesions was different from that of patients with simple contusion or SDH. All patients with combined lesions had broader traumatic areas, which established the conditions for diffuse swelling. After a great volume of SDH was evacuated, the majority of these patients showed a sharp decline in ICP over a short time. We infer that the drastic change of intracranial microenvironment would impair the stability of autoregulation. Without the force of extraparenchymal haematomas oppression, the original contusion and oedema would be more easily aggravated in subsequent treatments under impaired autoregulation [37, 38]. As reported in many previous studies, motor scores, midline shift and compressed basal cisterns were the most powerful independent risk factors for predicting the outcome [19, 29]. In this study, we did not find any significant correlation between these factors and SDC requirement. This indicated that traditional clinical severity indicators or CT parameters should not be simply used to predict the risk for the need of SDC after craniotomy.

### Limitations of the study

This study involved a retrospective analysis in a single centre. With respect to the inclusion criteria, for better control of the eligible population, we excluded patients with severe extracranial injury, abnormal coagulation and those aged >65 years. However, these variables may further affect the progression of postoperative intracranial hypertension as a risk factor of SDC requirement. Furthermore, we observed that Nagelkerke R^2^ (0.311) of the multivariate model was not very strong. This could be related to the study purpose. To completely explain the risk for the need of SDC, postoperative variables such as epilepsy and delayed hypotension should have been evaluated. In this study, we wanted to investigate whether the preoperative data can provide valuable clues about the risk for re-operation before making the decision about bone flap disposal. The results indicate that initial ICP has the potential to be used an indicator.

## Conclusion

Our study demonstrates that preoperative data can be used to evaluate the treatment options for STBI. Among the patients with STBI but without intraoperative brain swelling after mass lesion evacuation by craniotomy, the incidence of re-operation using SDC was 14.4 %. We found that initial ICP was an independent risk factor for re-operation. We also found that there was a significant correlation between SDC requirement and older age, early hypotension and combined lesions. Our results present the statistical inference of clinical data. To further validate the conclusions, a randomised, controlled trial should be performed.

## Abbreviations

SDC: Salvage decompressive craniectomy
ICP: Intracranial pressure
STBI: Severe traumatic brain injury
SDH: Subdural haematoma
DC: Decompressive craniectomy
CPP: Cerebral perfusion pressure
GCS: Glasgow Coma Scale
ROC: Receiver operating characteristic
GOSE: Extended Glasgow Outcome Score
